# Dermal Substitutes Support the Growth of Human Skin-Derived Mesenchymal Stromal Cells: Potential Tool for Skin Regeneration

**DOI:** 10.1371/journal.pone.0089542

**Published:** 2014-02-26

**Authors:** Talita da Silva Jeremias, Rafaela Grecco Machado, Silvia Beatriz Coutinho Visoni, Maurício José Pereima, Dilmar Francisco Leonardi, Andrea Gonçalves Trentin

**Affiliations:** 1 Departamento de Biologia Celular, Embriologia e Genética, Centro de Ciências Biológicas, Universidade Federal de Santa Catarina, Florianópolis, Santa Catarina, Brasil; 2 Departamento de Pediatria, Centro de Ciências da Saúde, Universidade Federal de Santa Catarina, Florianópolis, Santa Catarina, Brasil; 3 Hospital Infantil Joana de Gusmão, Florianópolis, Santa Catarina, Brasil; 4 Hospital Governador Celso Ramos, Florianópolis, Santa Catarina, Brasil; 5 Departamento de Cirurgia, Universidade do Sul de Santa Catarina, Florianópolis, Santa Catarina, Brasil; Wake Forest Institute for Regenerative Medicine, United States of America

## Abstract

New strategies for skin regeneration are needed in order to provide effective treatment for cutaneous wounds and disease. Mesenchymal stem cells (MSCs) are an attractive source of cells for tissue engineering because of their prolonged self-renewal capacity, multipotentiality, and ability to release active molecules important for tissue repair. In this paper, we show that human skin-derived mesenchymal stromal cells (SD-MSCs) display similar characteristics to the multipotent MSCs. We also evaluate their growth in a three-dimensional (3D) culture system with dermal substitutes (Integra and Pelnac). When cultured in monolayers, SD-MSCs expressed mesenchymal markers, such as CD105, Fibronectin, and α-SMA; and neural markers, such as Nestin and βIII-Tubulin; at transcriptional and/or protein level. Integra and Pelnac equally supported the adhesion, spread and growth of human SD-MSCs in 3D culture, maintaining the MSC characteristics and the expression of multilineage markers. Therefore, dermal substitutes support the growth of mesenchymal stromal cells from human skin, promising an effective tool for tissue engineering and regenerative technology.

## Introduction

Full-thickness skin injuries, such as extensive burns, chronic ulcers and deep wounds result in numerous physiological and functional problems. Their treatment requires a coverage that supports repair and restoration of skin functionality. Traditional procedures use autologous skin grafts for that purpose. However, removal of patients’ healthy skin is a highly invasive procedure and impossible to perform in some cases, such as extensive burns [1]. Several efforts in the field of tissue engineering have been made in order to develop a more effective and feasible treatment [2]. Such studies aim to identify molecules involved in tissue repair and to develop biomaterials that resemble the skin tissue architecture for cell therapy [3]. Notably, dermal substitutes have been used to cover the lesion to facilitate cell colonization, thereby promoting dermal regeneration [4]. Commercially available dermal substitutes have been developed with different matrices, including the dermal regeneration template Integra (Integra Lifescience, Plainsboro, NJ, USA) and Pelnac (Gunze Limited, Kyoto, Japan). Integra is a bilaminar membrane with an external silicone layer, which simulates epidermal function, and an inner layer consisting of bovine collagen fibers attached to chondroitin-6-sulfate glycosaminoglycan (shark cartilage-derived) with a mean pore diameter of 80 µm [4]. Similarly, Pelnac matrix is a bilaminar membrane with an external silicone layer and a porcine collagen sponge matrix with pore diameter in the range of 60–110 µm [5]. In both matrices, the inner layer (dermal layer) serves as a scaffold for vascularization and colonization by dermal fibroblasts [4]; [5].

Recent cell-based therapies for cutaneous lesions have combined bioartificial scaffolds and stem cells in order to improve skin regeneration [6]. In this scenario, mesenchymal stem cells (MSCs) provide several advantages, such as multipotentiality and the ability to expand *in vitro* for long periods [7]. MSCs constitute a population of adherent cells with fibroblast-like morphology and self-renewal capacity and the potential to differentiate into osteocytes, chrondrocytes and adipocytes [8]; [9]. These cells are also characterized by the expression of a specific pattern of cell-surface markers, both positive, including CD105, CD73 and CD90, and negative, including CD45, CD34, and CD14 or CD11b [9]. Moreover, MSCs modulate immune and inflammatory responses, and they also release active molecules that affect cell migration, proliferation and survival at the site of lesion. Therefore, MSCs play an active role in inflammatory, proliferative and remodeling phases of skin regeneration, thus improving tissue repair [10]; [11]. Importantly, stromal cells with functional and phenotypic proprieties similar to MSCs have been identified in skin [12].

It has been suggested that the combination of stem or progenitors cells with synthetic or natural scaffolds can provide an improved microenvironment for cell survival and functions compared with the inoculation of isolated cells directly at the site of lesion [13]. In this paper, we assessed the multipotent characteristics of human skin-derived mesenchymal stromal cells (SD-MSCs) cultured in monolayers and evaluated their integration with the dermal substitutes Integra and Pelnac through a three-dimensional (3D) culture system. Our results suggest that the association of human SD-MSC with dermal templates provides a cell-based therapeutic potential for skin regeneration.

## Materials and Methods

### Isolation and Culture of Human Skin-derived Mesenchymal Stromal Cells (SD-MSCs)

Human tissue fragments were obtained by written informed consent from healthy patients undergoing facial lifting. The procedure was approved by the Ethics Committee of the Federal University of Santa Catarina, Brazil. Tissue samples were digested with dispase (12.5 U/mL, 15 h, 4°C; BD), and the dermis was mechanically removed and digested again with 0.25% trypsin–EDTA (30 min, 37°C, Invitrogen). The obtained cell suspension was filtered through a 70-µm mesh (BD), centrifuged (500 g; 10 min; 22°C) and plated in 25-cm^2^ flasks (Corning) in standard medium consisting of Dulbecco’s Modified Eagle’s Medium-F12 (DMEM-F12; Invitrogen) supplemented with 15% fetal bovine serum (FBS; Vitrocell), penicillin (200 U/mL; Invitrogen) and streptomycin (10 mg/mL; Invitrogen). Cells were maintained until confluence at 37°C in a humidified 5% CO_2_ atmosphere with medium changed every 3 days and expanded up to 20 multiple passages.

### Culture of Human SD-MSCs on Dermal Substitutes

The dermal substitutes Integra and Pelnac were cut into fragments of 3–4 mm^2^ with a surgical punch, washed with PBS (10 min) and then with standard medium (10 min), followed by placement in 96-well culture plates with the dermal layer up. Cells (1×10^4/^well) were seeded and maintained as described above.

### Osteogenic Differentiation

Osteogenic differentiation was performed as previously described with some modifications [14]. Briefly, cells (3×10^4^/well in 24-well plates) were cultured in DMEM supplemented with dexamethasone (10^−9^ M; Sigma-Aldrich), ascorbate-2-phosphate (50 ug/mL; Sigma-Aldrich), b-glycerolphosphate (3.15 mg/mL; Sigma-Aldrich), 10% FBS and antibiotics. The medium was changed every 3 days. Control cells were cultured in standard medium. After 30 days, cells were fixed in 4% paraformaldehyde (Sigma-Aldrich) and stained with 2% Alizarin Red (Sigma-Aldrich) solution for 5 min.

### Adipogenic Differentiation

Adipogenic differentiation was performed as previously described (Coura et al. 2008). Briefly, cells (3×10^4^/well in 24-well plates) were cultured in DMEM supplemented with dexamethasone (10^−8 ^M; Sigma-Aldrich), indomethacin (100 uM; Sigma-Aldrich), insulin (2.5 ug/mL; Sigma-Aldrich), 10% FBS and antibiotics. The medium was changed every 3 days. Control cells were cultured in standard medium. After 30 days, cells were fixed in 4% paraformaldehyde and stained with 2% Oil Red O (Sigma-Aldrich) solution for 5 min.

### Flow Cytometry

Isolated skin cells (10^5^ cells) were harvested by trypsinization and incubated (60 min; 4°C) with the following antibodies (all from BD Bioscience): fluorescein isothiocyanate (FITC)-conjugated anti-CD73 or -CD45; phycoerythrin (PE)-conjugated anti-CD90 or peridin chlorophyll protein (PerCP)-conjugated anti-CD-105. Negative control staining was performed by using FITC-, PE- or PerCP-conjugated mouse IgG isotype antibodies. Cells were analyzed in a FACSCalibur flow cytometer (BD Bioscience), and data were examined by FLOWJO software.

### Immunofluorescence Staining

Cells were fixed in 4% formaldehyde, washed in PBS, and permeabilized for the analysis of intracellular markers (20 min, 0.25% Triton X-100; Sigma). The monolayers were then incubated with a blocking solution (PBS with 5% FBS) (45 min, room temperature), followed by incubation (overnight at 4°C) with the primary antibodies: anti-CD105 (Southern Biotech), anti-Nestin (Abcam), anti-α-SMA (α-smooth muscle actin, Sigma-Aldrich), anti-βIII-Tubulin (Promega) and anti-Fibronectin (Dako). After extensive washing in PBS, a second incubation (1 h; 37°C) with Alexa Fluor-488- or Alexa Fluor-547-specific anti-mouse or anti-rabbit secondary antibodies (all from Invitrogen) was performed. Cell nuclei were stained with 40, 6-diamino-2-phenylindole (DAPI; Sigma-Aldrich). Florescence labeling was observed using an epifluorescent microscope (Olympus IX71).

### Proliferation and Viability Assay (MTS Assay)

Cell proliferation and viability were analyzed by the CellTiter 96 AQueous Non-Radioactive Cell Proliferation Assay (Promega) according to the manufacturer’s instructions. Briefly, cells (1×10^4^/well in a 96-well plate) were seeded on dermal substitutes or on the plastic surface, and after 1, 4 and 7 days, the culture was incubated with MTS/PMS solution diluted in standard medium (4 h, 37°C, humidified 5% CO_2_ atmosphere). The formazan product was quantified by absorbance at 490 nm in a microplate reader (Tecan Infinite M200).

### Confocal Microscopy

Three days after cell seeding on dermal substitutes, cultures were fixed in 4% paraformaldehyde for 30 min, stained with DAPI and visualized under confocal microscopy (Leica DMI6000). Three-dimensional reconstruction of the scaffold was performed using ImageJ software.

### Scanning Electron Microscopy

Forty-eight hours after cell seeding on dermal substitutes, cultures were fixed (2.5% glutaraldehyde in 0.1 M sodium cacodylate buffer, Sigma-Aldrich) (12 h, 4°C), washed (sodium cacodylate buffer) and post-fixed (1% osmium tetroxide solution, 2 h). After dehydration (30%, 50%, 70%, 90% and 100% ethanol), cultures were dried in a critical point of CO_2_ (Leica MS CPD 030), metalized with 30 nm gold overlay (Leica EM SCD 500), and analyzed in a scanning electron microscope (Jeol JSM-6390LV) under capturing electrons at 15 kV by side illumination.

### Reverse Transcription-Polymerase Chain Reaction

Total RNA was isolated using the TRIZOL reagent following the manufacturer’s instructions (Invitrogen). Samples were then treated with DNase RQ1 RNase Free (Promega), according to the manufacturer’s instructions, to avoid any contaminating DNA. The RNA was quantified by spectrophotometry, and 2 µg of RNA were used for reverse transcription using the Thermoscript RT-PCR system for first-strand cDNA synthesis (Invitrogen). The PCR reactions were done using 1 µL of the RT reaction mixture and specific oligonucleotide primers for, GAPDH (glyceraldehyde-3-phosphate dehydrogenase), Nestin, βIII-Tubulin, α-SMA, CD31 and CD90. Oligonucleotide sequences and PCR conditions are shown in Table S1. GAPDH expression was used as an internal control of RNA integrity and efficiency of the reverse transcription process. Seven microliters of the PCR mixture were separated by electrophoresis on 2% agarose gel, and the reaction products were visualized under ultraviolet-induced fluorescence. All experiments were performed in triplicate.

## Results

### Phenotypic Characterization of Human SD-MSCs

**Figure 1:**
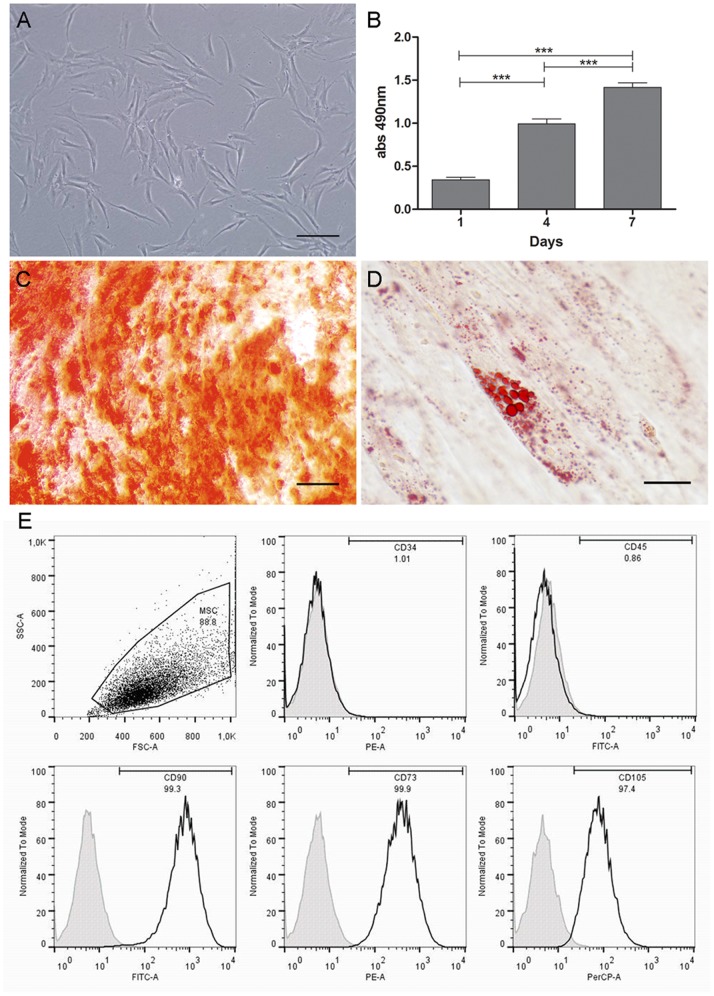
MSC phenotypic characterization of human skin-derived cells. (A) Morphological analysis of skin-derived cells by phase contrast microscopy. (B) MTS cell proliferation/viability assay. (C) Osteogenic and (D) adipogenic differentiation. (C) Cells cultured in inductive medium formed Alizarin Red S-stained mineralized nodules and (D) Oil red O-stained lipid clusters. (E) Flow cytometry analysis of hematopoietic (CD34, CD45) and MSC (CD90, CD73, CD105) markers. Specific markers are shown by black curves and controls by gray curves. ***p<0.001. Scale bar: (C–F): 50 µm. Other pictures: 200 µm.

Skin-derived cells with a fibroblast-like morphology adhered to the plastic culture dish (Figure 1A). Cells could be maintained in this condition for at least 20 passages. The high expansion capacity *in vitro* was confirmed by the progressive increase in the values of MTS assay during the 7 days of culture (Figure 1B).

The mesenchymal stem cell (MSC) characteristics were evaluated by the ability to originate osteogenic (Figure 1C), adipogenic (Figure 1D) and chondrogenic (data not shown) phenotypes. Skin-derived cells upon induction, were capable of differentiating into osteoblasts and adipocytes, characterized by dense extracellular matrix with calcium deposition (Figure 1C), and intracellular lipids (Figure 1D), respectively; nevertheless strong chondrogenic differentiation (data not shown) could not be observed, as previously reported [15].

Following MSC characterization, the expression of hematopoietic (CD34 and CD45) and mesenchymal (CD90, CD73 and CD105) stem cell markers was evaluated by flow cytometry (Figure 1E). Skin-derived cells were negative for CD34 and CD45 and positive for CD90 (99.3%), CD73 (99.9%) and CD105 (97.4%). The results were similar at both low (P1–P4) and high passages (P10–P20) (data not shown). Therefore, human skin-derived cells display such MSC characteristics as plastic adhesion, fibroblast morphology, mesodermal differentiation capacity, expression of mesenchymal markers, and absence of hematopoietic markers. Thereafter, these cells were termed skin-derived mesenchymal stromal cells (SD-MSCs).

### Expression of Multilineage Markers

**Figure 2:**
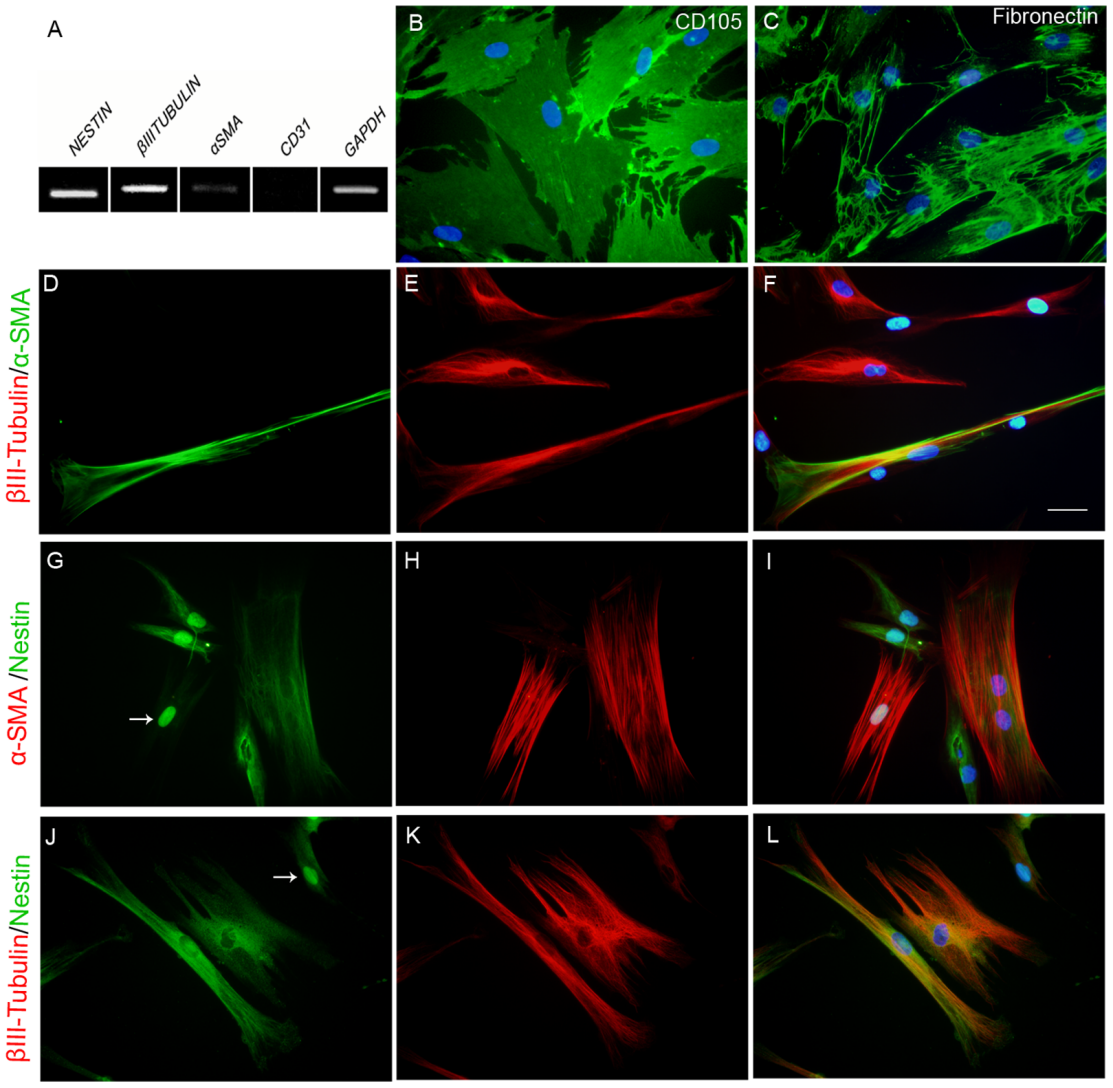
Multilineage potential of SD-MSCs. (A) Gene expression profile by RT-PCR of SD-MSCs cultivated in standard medium. (B–E) Immunofluorescence staining of (B) CD105 and (C) Fibronectin. (D–F) Double-staining of α-SMA and βIII-Tubulin, (G–I) α-SMA and Nestin and (J–L) βIII-Tubulin and Nestin co-expression. (F, I and L): Merged pictures of D–E, G–H, J–K, respectively. Cell nuclei were stained with DAPI (blue). Arrows: Nestin nuclear staining. Scale bar: 50 µm.

Next, the expression of markers of mesenchymal (α-SMA, CD105 and Fibronectin), neural (Nestin and βIII-Tubulin), and endothelial cells (CD31) by SD-MSCs was investigated (Figure 2). RT-PCR assay demonstrated the mRNA expression of Nestin, βIII-Tubulin and α-SMA, but not CD31 (Figure 2A). At the protein level, as revealed by immunofluorescence staining, most SD-MSCs expressed CD105 (Figure 2B), Fibronectin (Figure 2C), βIII-Tubulin (Figure 2E-F and K–L) and Nestin (Figure 2 G-I and J–L). Surprisingly, a nuclear distribution of Nestin was visualized in some cells (arrows in Figure 2G and J). In addition, about 40% of cells were positive to α-SMA (Figure 2D–H). Co-expression of Nestin, βIII-Tubulin and α-SMA is shown in Figure 2D–L.

### Viability/proliferation of Human SD-MSCs in 3D Culture with Dermal Substitutes

**Figure 3:**
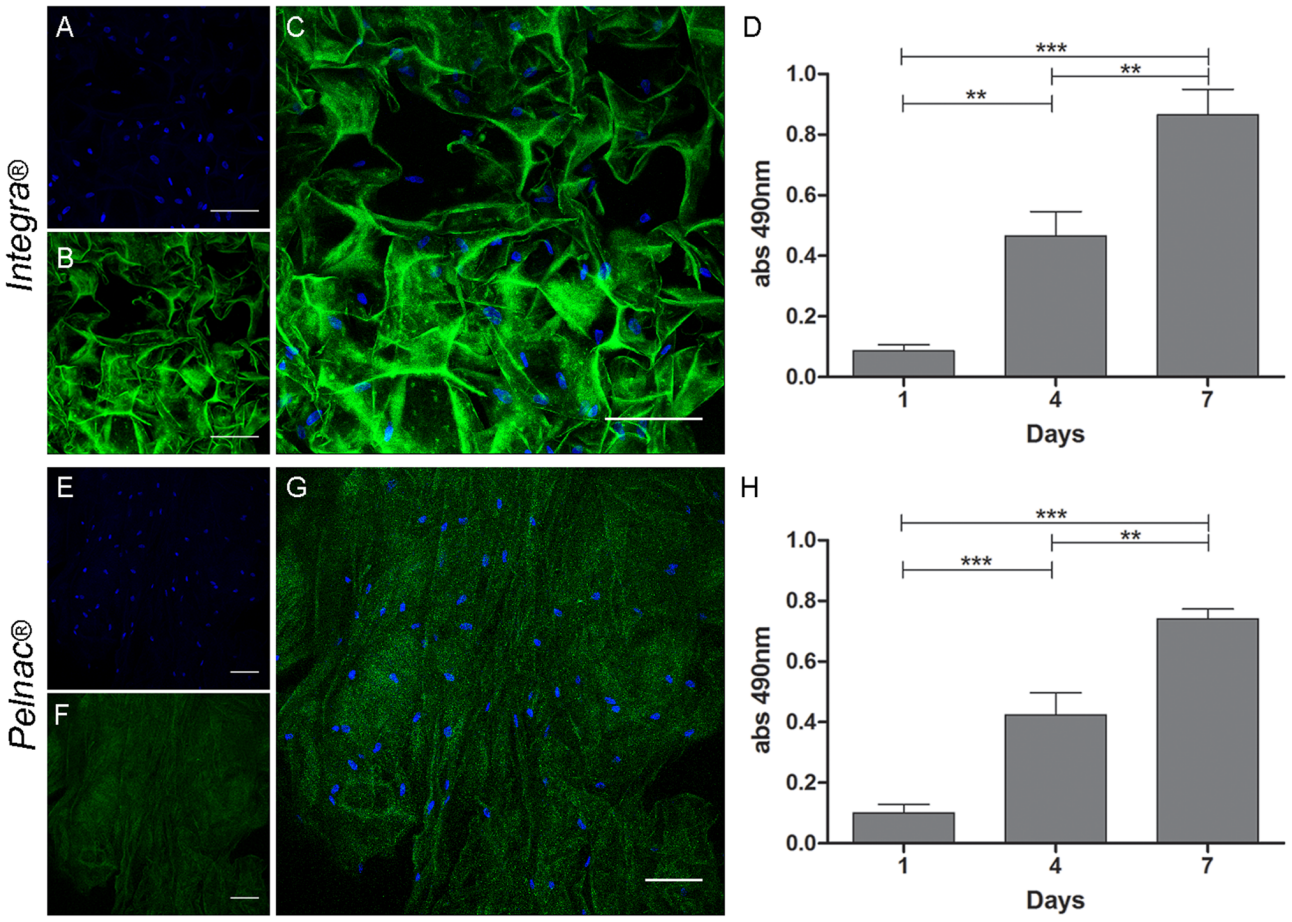
3D cultures of human SD-MSCs in (A–D) Integra and (E–H) Pelnac. Confocal microscopy of SD-MSCs cultured in (A–C) Integra and (D–F) Pelnac. (A and E) DAPI nuclear staining of SD-MSCs. (B and F) Integra and (F) Pelnac dermal substitutes, respectively (green autofluorescence). (C and G) merged images of (A and B) and (E and F), respectively. (D and H) MTS cell viability assay of SD-MSCs cultivated in Integra and Pelnac, respectively. ***p<0.001, **p<0.01. Scale bar: 100 um.

The ability of the dermal substitutes Integra and Pelnac to support the survival and growth of SD-MSCs was assessed by MTS assay. Significant and progressive increase in viability/proliferation of SD-MSCs with both dermal substitutes was detected during the 7-day period of culture (Figure 3D and H for Integra and Pelnac, respectively). Differences (P>0.05) in the MTS values between Integra and Pelnac were not observed at any time point of the analysis.

### Adhesion and Morphology of Human SD-MSCs in 3D Culture with Dermal Substitutes

**Figure 4:**
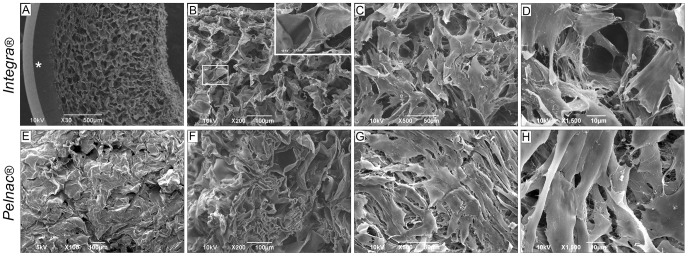
Scanning electron microscopy (SEM) images of SD-MSCs cultured in (A–D) Integra and (E–H) Pelnac. (A) Cross-sectional view of Integra dermal substitute alone showing the silicone (*) and the inner layer. (B–D) 3D culture of SD-MSCs in Integra 48 hours after seeding. (E) Surface and (F) cross-sectional views of Pelnac showing the collagen layer. (G–H) 3D culture of SD-MSCs in Pelnac 48 hours after seeding. Insets in B: different magnifications of a SD-MSC.

Confocal microscopy analysis revealed that SD-MSCs adhere and migrate within both Integra (Figure 3A–D) and Pelnac (Figure 3 E–H) matrices, although mostly distributed in the upper portion (Figure S1). The structure and porosity of Integra (Figure 4A) and Pelnac (Figure 4E–F) inner layers were shown by scanning electron microscopy (SEM). SEM analysis also revealed that SD-MSCs similarly attached and spread at both dermal matrices with the fibroblast-like fusiform morphology (Figure 4B-D and G-H for Integra and Pelnac, respectively). Together with MTS assay, these findings indicate that both dermal substitutes equally support the adhesion, spread and growth of SD-MSCs *in vitro*.

### Phenotypic Profile of Human SD-MSCs in 3D Cultures with Dermal Substitutes

**Figure 5:**
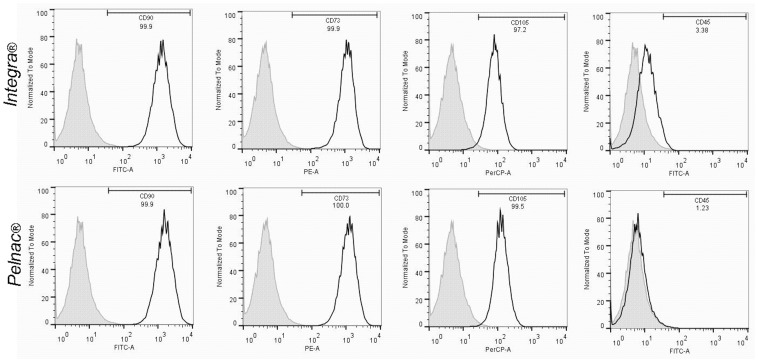
Immunophenotypic profile of human SD-MSCs cultured in Integra and Pelnac. Flow cytometry analysis of SD-MSCs (CD90, CD73, CD105) and hematopoietic (CD45) markers in SD-MSCs after 3 days of culture in Integra (upper panel) and Pelnac (lower panel). Curves in black show the specific markers, and gray curves correspond to controls.

**Figure 6:**
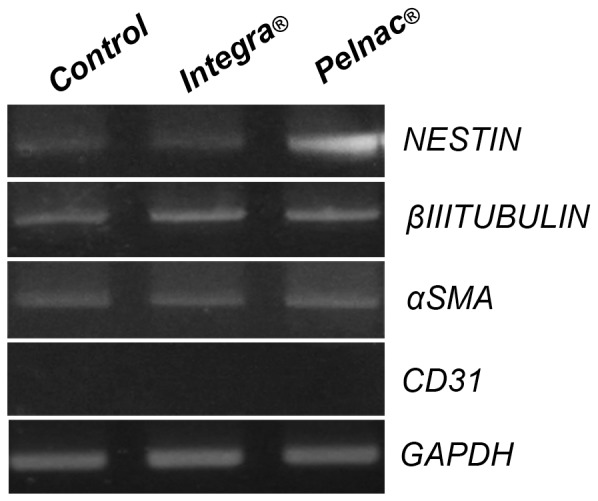
Gene expression profile of human SD-MSCs cultured in Integra and Pelnac. RT-PCR analysis showing mRNA expression of Nestin, βIII-Tubulin, α-SMA, CD31 and GAPDH (as internal control) in SD-MSCs cultured in dermal substitutes. Control: SD-MSCs cultured on plastic surfaces.

Most SD-MSCs cultured within Integra (Figure 5, upper panel) and Pelnac (Figure 5, lower panel) matrices were positive to the CD90 (99.9% and 99.9%, respectively), CD73 (99.9%, and 100%, respectively) and CD105 (97.2% and 99.5%, respectively) markers, but negative to CD45 (less than 3.38%) by flow cytometry. Consequently, SD-MSCs expressed the mRNA of Nestin, βIII-Tubulin, and α-SMA, but not the CD31 mRNA (Figure 6). Therefore, SD-MSCs in 3D culture with both Integra and Pelnac matrices are able to maintain phenotypic characteristics similar to MSCs.

## Discussion

In this study, mesenchymal stromal cells were isolated from human skin. These skin-derived mesenchymal stromal cells share the criteria established by the International Society of Cellular Therapy for Human MSC [9] including: adhesion to plastic culture dish, fibroblast morphology, adipogenic and osteogenic differentiation, expression of mesenchymal markers and absence of hematopoietic markers. Indeed, stromal cells have been demonstrated to exhibit MSC characteristics such as cell phenotype, transdifferentiation potential and immunosuppressive properties and thus are functionally comparable to MSCs, being difficult to discriminate between them [12].

In addition, despite the absence of a neuron-like morphology, most SD-MSCs expressed the neural proteins Nestin [16]; [17] and βIII-Tubulin [18]. Both were also reported in MSCs [19] [20]; [21]. Interestingly, similar to some brain tumor cell lines [16], we detected in some SD-MSCs the presence of Nestin by nuclear staining, which suggests the possible involvement of gene expression. Therefore, a possible neural potential of human SD-MSCs could be implicated. Future studies are needed to determine the role of these cytoskeleton proteins in SD-MSCs [22].

In our study, about 40% of SD-MSCs were positive to α-SMA, a cytoskeleton marker of smooth muscle cells, also related to vasculogenesis [23]. In fact, smooth muscle markers have been reported in MSCs with multilineage potential [24] which could be related to the multipotentiality of these cells. Efforts to track the identity of tissue-resident MSCs have suggested their perivascular origin [25]; [7], evidenced by the pericyte-like features [26]. Moreover, the three cytoskeletal proteins, α-SMA, Nestin and βIII-Tubulin, which we found in human SD-MSCs were also observed in pericytes [27]; [28] [29], suggesting a possible perivascular origin.

### Dermal Substitutes Integra and Pelnac Allow Survival, Adhesion and Spreading of Human SD-MSCs in a 3D Culture System

The combination of stem cells with scaffolds has been used with some success in several injuries [30]. In this paper, we investigated *in vitro* the feasibility and efficacy of combining human SD-MSCs with dermal substitutes for clinical use. The biocompatibility of Integra and Pelnac with SD-MSCs was demonstrated by 3D imaging and MTS assay that showed the infiltration, distribution and proliferation of these cells into both matrices. Although the cells were mostly concentrated in the upper portion of the matrices, possibly as a result of the top-down cell-seeding procedure, we believe that a uniform distribution could be achieved in the scaffolds by improving the technique. In addition, SD-MSCs could adhere to both matrices, establishing strong connections and cytoplasmic extensions and maintaining the typical MSC-fibroblast morphology, as revealed by SEM. Similar results were reported in human MSCs from bone marrow and adipose tissue, which were able to grow and proliferate in Integra [31]; [32]. Importantly, although Pelnac is largely used in Japan, its combination with MSCs was first demonstrated in the present study. Together, these observations confirmed that both dermal substitutes are suitable substrates for the colonization of SD-MSCs.

### Human SD-MSCs Maintain MSC Characteristics in 3D Culture with Integra and Pelnac Dermal Substitutes

It is well established that the composition and mechanical properties of the extracellular environment regulate intracellular signaling and thus influence different aspects of cell behavior, including cell growth, migration, survival and phenotypic fate [33]; [34]. Therefore, the effective attachment and maintenance of stem cells onto scaffolds is a critical step to be considered in tissue engineering [35]. Hence, to further investigate the efficiency of Integra and Pelnac in supporting the growth and maintenance of SD-MSCs, their phenotypic profile in 3D culture was studied. In fact, in this culture condition, most SD-MSCs were positive for the MSC markers CD105, CD73 and CD90, but negative for the hematopoietic stem cell marker CD45. In addition, they also expressed neural (Nestin and βIII-Tubulin) and mesenchymal (α-SMA) markers, but did not express the endothelial marker CD31. These results suggest that the phenotypic profile of SD-MSCs observed in conventional 2D plastic culture (monolayer) was maintained in a 3D culture system with dermal substitutes.

Despite differences in the composition of Integra and Pelnac, as explained above, our findings, when taken together, show that the growth and phenotypic characteristics of SD-MSCs were similar in both templates. Therefore, both dermal substitutes are biocompatible scaffolds for SD-MSCs growth, and the 3D culture system reported here represents a potential tool for tissue engineering.

## Conclusions

In this study, we isolated and characterized a population of cells from human skin termed herein as skin-derived mesenchymal stromal cells (SD-MSCs) with similar characteristics to MSCs. These cells express *in vitro* multilineage markers (mesenchymal and neural, but not hematopoietic or endothelial). We developed a 3D culture system of human SD-MSCs with the dermal substitutes Integra and Pelnac, in which these cells were found to adhere and proliferate, while maintaining the MSC properties and gene expression of multilineage markers. In conclusion, 3D culture systems using dermal substitutes described in this study provide an efficient *in vitro* environment for human SD-MSCs and could therefore be useful for tissue engineering and cell therapy.

## Supporting Information

Figure S1 **3D reconstruction of confocal images of human SD-MSC cultures in Integra and Pelnac.** (A) SD-MSCs cultured in Integra and (B) Pelnac. Blue: DAPI nuclear staining of SD-MSCs. Green: autofluorescence of dermal substitutes.
(TIF)

Table S1 **RT-PCR Conditions: Oligonucleotide primer set and amplified size.**
(DOCX)
