# Supplementary figures and images for: Dermal Substitutes Support the Growth of Human Skin-Derived Mesenchymal Stromal Cells: Potential Tool for Skin Regeneration
Source: PLoS One. 2014 Feb 26;9(2):e89542. doi: 10.1371/journal.pone.0089542 (PMC3935879; doi:10.1371/journal.pone.0089542)

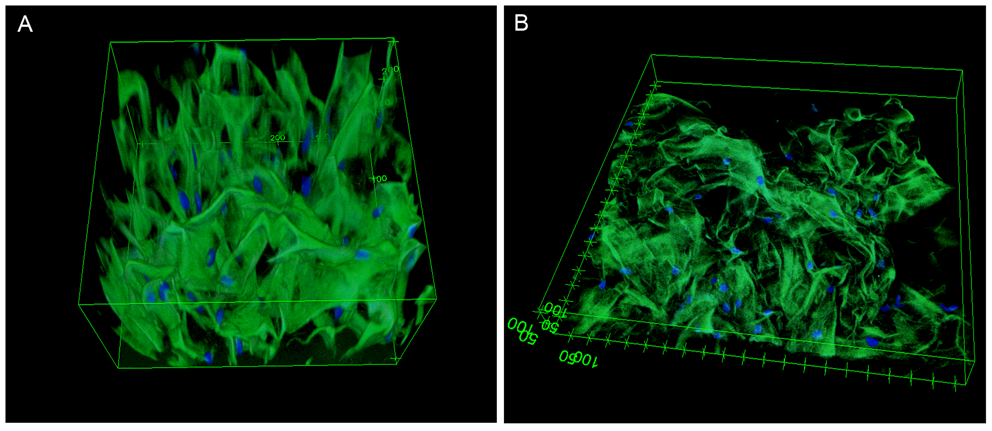

Supplement: Figure S1 — 3D reconstruction of confocal images of human SD-MSC cultures in Integra and Pelnac. (A) SD-MSCs cultured in Integra and (B) Pelnac. Blue: DAPI nuclear staining of SD-MSCs. Green: autofluorescence of dermal substitutes. (TIF) [file pone.0089542.s001.tif]
