# Supplementary material for: Dermal Substitutes Support the Growth of Human Skin-Derived Mesenchymal Stromal Cells: Potential Tool for Skin Regeneration
Source: PLoS One. 2014 Feb 26;9(2):e89542. doi: 10.1371/journal.pone.0089542 (PMC3935879; doi:10.1371/journal.pone.0089542)
Supplement: Table S1 — RT-PCR Conditions: Oligonucleotide primer set and amplified size. (DOCX) [file pone.0089542.s002.docx]

**Table S1. RT-PCR Conditions: Oligonucleotide primer set and amplified size**

| ***Gene*** | ***Oligonucleotide sequences*** | ***Size of amplified fragment (bp*)*** |
| --- | --- | --- |
| *αSMA* | Forward: CGATAGAACACGGCATCATC  Reverse: CATCAGGCAGTTCGTAGCTC | 522 |
| *βIIITUBULIN* | Forward:AGATGTACGAAGACGACGAGGAG  Reverse:GTATCCCCGAAAATATAAACACAAA | 312 |
| *CD31* | Forward:GAGTCCTGCTGACCCTTCTG  Reverse: ATTTTGCACCGTCCAGTCC | 107 |
| *NESTIN* | Forward:AGGGAAGTTGGGCTCAGGACTGG  Reverse:CAGCTGGCGCACCTCAAGATG | 208 |
| *GAPDH* | Forward:ATCACTGCCACCCAGAAGAC  Reverse:ATGAGGTCCACCACCCTGTT | 441 |

*α*SMA, alpha-smooth muscle actin; GAPDH, glyceraldehyde-3-phosphate dehydrogenase.* bp: base pairs
